# Purinergic signalling in the rostral ventro-lateral medulla controls sympathetic drive and contributes to the progression of heart failure following myocardial infarction in rats

**DOI:** 10.1007/s00395-012-0317-x

**Published:** 2012-11-28

**Authors:** Nephtali Marina, Feige Tang, Melina Figueiredo, Svetlana Mastitskaya, Vitaliy Kasimov, Vidya Mohamed-Ali, Eva Roloff, Anja G. Teschemacher, Alexander V. Gourine, Sergey Kasparov

**Affiliations:** 1 Neuroscience, Physiology and Pharmacology, University College London, London, WC1E 6BT UK; 2 School of Physiology and Pharmacology, Medical Sciences Building, Bristol Heart Institute, University of Bristol, Bristol, BS8 1TD UK

**Keywords:** Heart failure, Sympathetic nervous system, Medulla oblongata, Purinergic signalling, Viral gene transfer

## Abstract

Heart failure may lead to hypoperfusion and hypooxygenation of tissues and this is often exacerbated by central and obstructive sleep apnoeas associated with recurrent episodes of systemic hypoxia which triggers release of ATP within the CNS circuits controlling sympathetic outflow. Using in vitro and in vivo models we tested two hypotheses: (1) activated brainstem astroglia release ATP and via release of ATP activate sympathoexcitatory neurones of the rostral ventrolateral medulla (RVLM); and (2) ATP actions in the RVLM contribute to sympathoexcitation, progression of left ventricular (LV) remodelling and development heart failure secondary to myocardial infarction. In vitro, optogenetic activation of RVLM astrocytes transduced to express light-sensitive channelrhodopsin-2 activated sympathoexcitatory RVLM neurones in ATP-dependent manner. In anaesthetised rats in vivo, similar optogenetic activation of RVLM astrocytes increased sympathetic renal nerve activity, arterial blood pressure and heart rate. To interfere with ATP-mediated signalling by promoting its extracellular breakdown, we developed a lentiviral vector to express an ectonucleotidase—transmembrane prostatic acid phosphatase (TMPAP) on the cellular membranes. In rats with myocardial infarction-induced heart failure, expression of TMPAP bilaterally in the RVLM led to lower plasma noradrenaline concentration, maintained left ventricular end diastolic pressure, attenuated decline in d*P*/d*T*_max_ and shifted the LV pressure–volume relationship curve to the left. These results show that activated RVLM astrocytes are capable of increasing sympathetic activity via release of ATP while facilitated breakdown of ATP in the RVLM attenuates the progression of LV remodelling and heart failure secondary to myocardial infarction.

**Electronic supplementary material:**

The online version of this article (doi:10.1007/s00395-012-0317-x) contains supplementary material, which is available to authorized users.

## Introduction

Congestive heart failure is associated with an increased activity of the sympathetic nervous system. It is generally believed that increased sympathetic tone is maladaptive and detrimental, contributing to the progression of the disease [5, 26]. There is evidence that sleep-disordered breathing may contribute to sympathoexcitation in heart failure. 27 % of heart failure patients have obstructive sleep apnoea and 38 % have central sleep apnoea [3, 4]. Heart failure patients with sleep apnoea have higher sympathetic tone compared to patients with normal breathing patterns [30, 32]. Alterations in the central nervous mechanisms of sympathetic control may potentiate those triggered by the modified afferent input from the affected myocardium [22] exacerbating progression of heart failure.

Sympathetic activity is generated by several distinct neuronal populations in the CNS, including regions of the rostral ventrolateral medulla oblongata (RVLM) believed to provide a major descending monosynaptic excitatory drive to spinal sympathetic preganglionic neurones [6, 7, 20, 21, 29, 31, 40, 42]. Sympathoexcitatory RVLM neurones include two main cell groups, a subgroup of medullary C1 catecholaminergic neurones and a population of non-catecholaminergic, possibly glutamatergic neurones [20, 21, 29, 41, 42]. These sympathoexcitatory RVLM neurones, often referred to as presympathetic neurones, respond to changes in *P*O_2_ and may contribute to the increases in sympathetic cardiac and vasomotor activities during systemic hypoxia [27, 28, 39]. In support of this hypothesis, RVLM neurones were found to be activated during the development of heart failure in an animal model using c-fos immunohistochemistry [18].

The exact mechanisms of oxygen-sensitivity of the RVLM neurones are not known, but there is evidence suggesting that it may be mediated by prior release of ATP. First, systemic hypoxia triggers marked release of ATP from the ventral medullary regions located within and in a close proximity to the RVLM [16]. Second, presympathetic RVLM neurones are highly sensitive to ATP. They are excited in response to application of exogenous ATP or ATP receptor agonists [37, 43], while activation of ATP receptors in the RVLM evokes marked increases in the arterial blood pressure, heart rate and renal sympathetic nerve activity [23, 37, 43, 44]. Taken together these data suggest that in heart failure hypoperfusion and hypooxygenation of brain tissues (often exacerbated by central and obstructive sleep apnoeas) may result in an increase in extracellular concentration of ATP in the RVLM leading to higher level of activity of sympathoexcitatory neurones and increased central sympathetic drive.

Here, we tested this hypothesis using in vitro and in vivo rat models in combination with viral gene transfer and optogenetic approaches. Optogenetic activation of mammalian cells requires expression of one of the light sensitive actuator proteins originating from algae, such as channelrhodopsin-2 (ChR2). ChR2 is sensitive to blue light and when activated and opens a pore permeable to Na^+^, K^+^, H^+^ and Ca^2+^. Optogenetics gives an opportunity of stimulation of a particular cell type (in our case astrocytes) with unprecedented resolution and specificity [45]. In our recent study we used this approach and found that glial cells, the astrocytes, which reside at the ventral surface of the medulla oblongata in so-called central chemosensitive area are exquisitely chemosensitive and respond to physiological increases in *P*CO_2_/[H^+^] with release of ATP [15]. Astrocytes in other brain areas are also well known to communicate via release of ATP and constitute one of the main sources of extracellular ATP in the brain. Moreover, astrocytes are capable of responding to hypoxic stimuli with Ca^2+^ elevations [1], which could lead to the release of ATP. Based on these reasons, we first determined whether activated brainstem astrocytes are able to excite RVLM presympathetic neurones via release of ATP (in vitro) and enhance vasomotor sympathetic activity (in vivo). Then, we determined whether ATP actions in the RVLM contribute to pathological sympathoexcitation, progression of left ventricular (LV) remodelling and heart failure developing after an acute myocardial infarction (MI) in rats.

## Materials and methods

All animal experiments were performed in accordance with the UK Home Office (Scientific Procedures) Act (1986) and associated guidelines.

### Optogenetic activation of RVLM astrocytes in vitro

Signalling mechanisms between astrocytes and sympathoexcitatory C1 neurones were studied in organotypic slices of the rat brainstem prepared as described in detail previously [14, 19]. To identify C1 neurones, slice cultures were transduced at the time of plating with an adenoviral vector with PRS×8 promoter driving the expression of a fluorescent protein DsRed2 [19]. For activation of astrocytes using light, slices were co-transduced 4–5 days later with another adenoviral vector AVV-sGFAP-ChR2(H134R)-Katushka1.3 where enhanced GFAP promoter is used to drive astroglial expression of a ChR2(H134R) variant fused with a far red-shifted fluorescent protein as previously described in detail in [15]. After 8–10 days in culture, the slices were transferred into a recording chamber mounted on a stage of Leica SP confocal microscope. Green laser light (532 nm) was used to identify C1 neurones expressing DsRed. Neurones were recorded in whole-cell patch clamp configuration, as described [15]. Blue light (470 nm, <1 mW) was used to activate astrocytes expressing ChR2(H134R).

### Stereotaxic delivery of viral vectors

Rats (Sprague–Dawley, 200–250 g) were anaesthetised with a mixture of ketamine (60 mg kg^−1^; i.m.) and medetomidine (250 μg kg^−1^, i.m.) and two microinjections per side (1 μl each, 0.1 μl min^−1^) of a viral suspension containing AVV-sGFAP-ChR2(H134R)-Katushka1.3 (titre 5 × 10^9^) or AVV-sGFAP-ChR2-Venus (titre 5 × 10^9^) were stereotaxically delivered bilaterally into the RVLM using the following coordinates from Bregma: 11 and 12 mm caudal, 2 mm lateral and 8.5 mm ventral. The wound was sutured and anaesthesia was reversed with atipamezole (1 mg kg^−1^, i.m.).

### Optogenetic activation of RVLM astrocytes in vivo

Rats transduced to express ChR2(H134R)-Katushka1.3 or ChR2-Venus in the RVLM astrocytes were anaesthetised with isoflurane (3 %) 10–12 days after microinjections of the viral vector into the RVLM. The femoral artery and vein were cannulated for monitoring arterial blood pressure and administration of anaesthetic, respectively. The depth of anaesthesia was monitored using stability of blood pressure, heart rate and lack of flexor responses to a paw-pinch. The ventral surface of the brainstem was exposed as described previously [16]. The trachea was cannulated and the animal was mechanically ventilated with O_2_-enriched (30 %) air. End-tidal level of CO_2_ was monitored continuously (Capstar-100, CWE Inc, USA) and arterial *P*CO_2_, *P*O_2_ and pH were measured regularly. The left renal nerve was dissected retroperitoneally and placed on bipolar silver electrodes. At the end of the preparative surgery α-chloralose (initial dose 100 mg kg^−1^ i.v., supplemented with 20 mg kg^−1^ i.v., as required) was infused whilst isoflurane was slowly withdrawn. The ventral medullary surface was superperfused with artificial cerebrospinal fluid (aCSF; 124 mM of NaCl, 3 mM KCl, 2 mM CaCl_2_, 26 mM NaHCO_3_, 1.25 mM NaH_2_PO_4_, 1 mM MgSO_4_, 10 mM d-glucose saturated with 95 % O_2_/5 % CO_2_, pH 7.4) and following a 30-min period of stabilisation illuminated unilaterally with blue laser light (445 nm; 20/20 ms duty cycle). Renal sympathetic nerve activity (SNA), arterial blood pressure and tracheal pressure were recorded using a Power1401 interface and *Spike2* software (Cambridge Electronic Design Ltd, UK). Changes in renal nerve discharge induced by optogenetic activation of RVLM astrocytes were normalised to resting activity during normoxic/normocapnic conditions (100 %) and no activity (0 %) recorded following administration of hexamethonium (20 mg kg^−1^; i.v.) at the end of each experiment.

### Generation and validation of a lentiviral vector for TMPAP expression in the brain

**Fig. 1:**
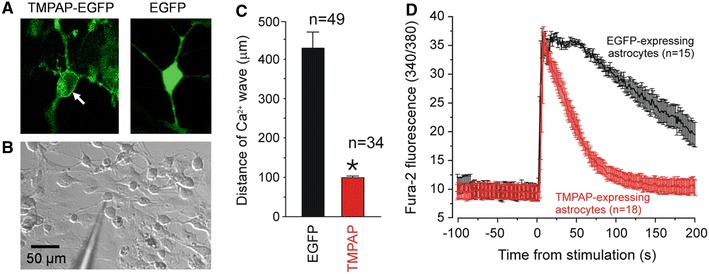
Transmembrane prostatic acid phosphatase (TMPAP) expression limits propagation of mechanical stimulation-induced Ca^2+^ wave in cultured astrocytes. **a** Cells in primary culture transduced with LVV-EF1α-TMPAP–EGFP or LVV-EF1α-EGFP. Note the predominant membrane localization of TMPAP–EGFP. **b** To trigger a ‘Ca^2+^ wave’, a single astrocyte in culture was mechanically stimulated with a patch pipette and [Ca^2+^]_i_ responses evoked in a stimulated and surrounding cells were recorded. **c** Bar graph (mean ± SEM) of the extent of mechanical stimulation-evoked Ca^2+^ wave propagation in astroglial cultures expressing EGFP and cultures transduced to express TMPAP–EGFP (*P < 0.001). Numbers of individual tests are indicated. **d** [Ca^2+^]_i_ responses evoked in individual-cultured astrocytes. [Ca^2+^]_i_ transients in astrocytes expressing EGFP and TMPAP were similar in amplitude, however, astrocytes transduced to express TMPAP displayed more rapid [Ca^2+^]_i_ recovery

Long-term application of pharmacological tools to study the role of ATP-mediated signalling in a defined area of the brain in chronic experiments is unfeasible. To interfere with ATP-mediated signalling by promoting extracellular breakdown of ATP, we developed a lentiviral vector (LVV) to express a potent ectonucleotidase—transmembrane prostatic acid phosphatase (TMPAP) under the control of an elongation factor 1α (EF1α) promoter [46]. To visualise transgene expression, C-terminus of TMPAP was fused with an enhanced green fluorescent protein (EGFP) (Fig. 1a). An established in vitro model was used to test the ability of LVV-EF1α-TMPAP–EGFP vector to drive the expression of TMPAP and interfere with ATP-mediated signalling. In response to mechanical stimulationm, astrocytes in cultures are known to generate ‘waves’ of Ca^2+^ excitation propagating via release of ATP [2, 33]. Primary astroglial cultures were prepared from the brains of P2 rat pups and transduced with either LVV-EF1α-TMPAP–EGFP or a control virus LVV-EF1α-EGFP. Astrocytes were loaded with a Ca^2+^ indicator Fura-2 and imaged using a Leica SP confocal microscope at 34 °C. To trigger a ‘Ca^2+^ wave’, a single astrocyte was mechanically stimulated with a patch pipette (Fig. 1b) and [Ca^2+^]_i_ responses evoked in a stimulated and the surrounding cells were recorded. To test the efficacy of TMPAP expression in facilitating breakdown of extracellular ATP, the spread of Ca^2+^ excitation was assessed by measuring the distance from the stimulated cell to the further-most responding cells in the area of stimulation (Fig. 1c) and the profile of Ca^2+^ responses in individual astrocytes was determined (Fig. 1d). TMPAP expression was found to dramatically reduce the spread of Ca^2+^ excitation propagating among cultured astrocytes in response to mechanical stimulation (Fig. 1c; *P* < 0.001; Student’s *t* test). [Ca^2+^]_i_ transients evoked by mechanical stimulation of astrocytes expressing EGFP (control) and TMPAP were similar in amplitude, however, astrocytes transduced to express TMPAP displayed rapid [Ca^2+^]_i_ recovery (Fig. 1d), likely due to a facilitated degradation of released ATP by TMPAP activity.

### Studying the effect of TMPAP expression in the RVLM on cardiovascular homeostasis in healthy freely behaving rats

Rats (250–300 g) were implanted with radio transmitters for chronic monitoring of the arterial blood pressure, as described earlier [8, 34]. Briefly, a blood pressure radio transmitter (model R46SP, Telemetry Research) was placed into the abdominal cavity and plumbed into the aorta under ketamine (60 mg kg^−1^; i.m.)/medetomidine (250 mg kg^−1^, i.m.) anaesthesia. Data were sampled using a Power1401 interface and *Spike2* software. Control measurements were taken 6–7 days after transmitter implantation for ~1 h every day for 2 weeks. Then, the animals were anaesthetised (ketamine/medetomidine) and a viral suspension containing LVV-EF1α-TMPAP–EGFP (1 × 10^10^ TU/ml) was microinjected bilaterally into the RVLM using stereotaxic coordinates indicated above. Therefore, the same animal served as its own control. The data acquisition began 7–10 days after LVV-EF1α-TMPAP–EGFP microinjections and continued for up to 7 subsequent days. Blood pressure and heart rate were sampled every first 10 min of every hour. Analysis was performed using a custom-made *Spike2* script following previously reported protocols [8, 34].

### Rat model of myocardial infarction-induced heart failure

MI was induced using the coronary occlusion technique described in detail elsewhere [11, 35, 36]. Briefly, rats 200–220 g (*n* = 50) were anaesthetised with a mixture of ketamine (60 mg kg^−1^; i.m.) and medetomidine (250 μg kg^−1^, i.m.), orotracheally intubated, and artificially ventilated with O_2_-enriched (30 %) air. A left thoracotomy was performed to expose the heart, the pericardium was opened and the heart was exteriorised. The left anterior descending coronary artery was ligated between the pulmonary outflow tract and the left atrium using a 5–0 Merisilk suture. Paleness of the anterior wall of the left ventricle (LV) confirmed successful coronary occlusion. Sham-operated rats were prepared in the same manner but did not undergo coronary ligation. The thoracic cavity was irrigated with 100–200 μl of a solution containing Penicillin (10,000 IU/ml) and Streptomycin (10,000 μg/ml) and the chest incision was closed. Anaesthesia was reversed with atipamezole (1 mg kg^−1^). Postoperative mortality within the 48 h after coronary occlusion was 35 % due to sudden cardiac death. Two days after MI or sham surgery, the rats were anaesthetised with a mixture of ketamine (60 mg kg^−1^; i.m.) and medetomidine (250 μg kg^−1^, i.m.) and LVV-EF1α-TMPAP–EGFP (1 × 10^10^ TU/ml) or LVV-EF1α-EGFP (1 × 10^10^ TU/ml), were microinjected bilaterally into the RVLM as described above.

### Haemodynamic measurements

Haemodynamic measurements were performed 6 weeks after MI or sham surgery. The animal was anaesthetised (urethane 1.5 g kg^−1^, i.p.), placed on a homeothermic blanket with body temperature kept at 37.0 ± 0.2 °C. The femoral artery and vein were cannulated and ECG electrodes were placed. To evaluate LV contractile function, LV pressure was recorded using a fibre optic pressure sensor (SA Instruments, USA) introduced into the LV chamber via the right carotid artery. Following a 30-min stabilisation period, the data were acquired using Power1401 interface, saved and analysed off-line using *Spike2* software. The maximum rate of rise of LV pressure (LV dP/dT_max_) was derived from the LV pressure recording using the slope function. Average waveforms (10-min data sets) were used to determine systolic blood pressure (SBP), diastolic blood pressure (DBP), LV systolic pressure (LVSP) and LV end diastolic pressure (LVEDP). At the end of the haemodynamic studies, the heart was arrested with KCl and removed. LV pressure–volume relationship curve was obtained using a double-lumen catheter as described in detail previously [10, 13].

### Quantification of the infarct size

The extent of myocardial infarction was determined as described in detail previously [36]. LV was frozen at −20 °C for 10 min and then sectioned from the apex to the base into four transverse slices of identical thickness (1.5 mm). In each slice, the length of the scar and noninfarcted muscle for endocardial and epicardial surfaces were determined by computerised planimetry. The ratio of the length of the scar and surface circumferences defined the infarct size for endo- and epicardial surfaces, respectively. Infarct size (%) was calculated as an average of infarcted endo- and epicardial surfaces.

### Determination of plasma noradrenaline levels

At the end of the haemodynamic study, 1.5 ml of the arterial blood was collected into an ice-cold tube containing 100 μl of EDTA-sodium metabisulfite solution (3.3 μg of sodium metabisulfite dissolved in 150 mL of a 0.1 M EDTA solution). Blood samples were gently mixed and immediately centrifuged at 5,000 rpm (20 min at 4 °C) followed by plasma collection and storage at −80 °C until assayed. Plasma noradrenaline concentration was determined by ELISA using a commercially available kit (Labor Diagnostika, Germany) according to manufacturer’s instructions.

### Statistical analysis

The data were analysed by ANOVA followed by the Tukey–Kramer’s post hoc test, Student’s *t* test, or non-parametric Wilcoxon-Mann–Whitney *U* test, as appropriate. Data are presented as either mean ± SEM or median and interquartile ranges. Differences between the experimental groups with *P* < 0.05 were considered significant.

## Results

### Optogenetic stimulation of brainstem astrocytes in vitro activates C1 RVLM neurones via an ATP-dependent mechanism

**Fig. 2:**
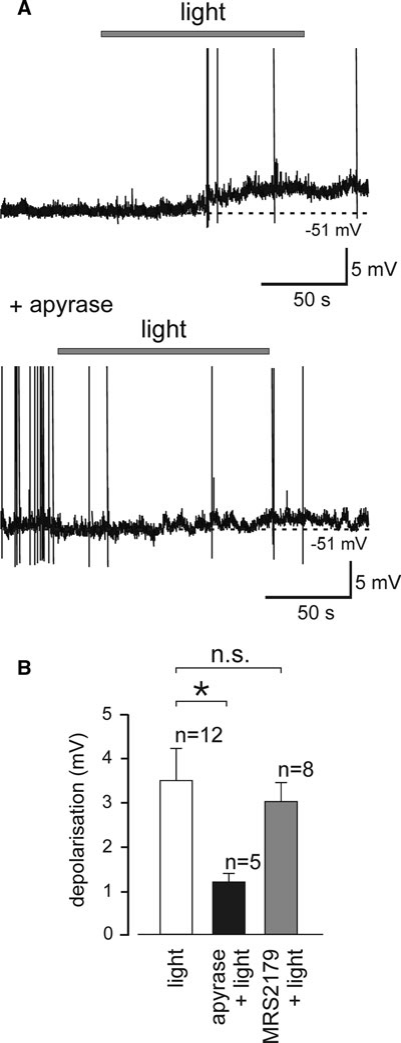
Optogenetic stimulation of RVLM astrocytes triggers ATP-dependent excitation of presympathetic C1 neurones. **a** Traces illustrate changes in membrane potential of two different C1 neurones from separate organotypic slices illustrating their responses to light activation of adjacent ChR2(H134R)-expressing astrocytes in the absence and presence of ATP diphosphohydrolase (apyrase, 25 U ml^−1^). Stimulation of astrocytes with flashing blue light (470 nm, 20/20 ms duty cycle for 120 s) led to depolarisation and firing of action potentials in C1 neurones. This effect was blocked in the presence of apyrase. Note that action potentials are truncated to illustrate changes in membrane potential. **b** Effects of apyrase and MRS2179 (10 μM) on changes in membrane potential of C1 neurones evoked by optogenetic stimulation of neighbouring astrocytes. Group data are shown as mean ± SEM. *P < 0.05

In organotypic brainstem slices, C1 neurones were targeted to express a fluorescent protein DsRed2 under the control of PRS×8 promoter while astrocytes were transduced to express ChR2(H134R). This allowed patch clamp recordings from C1 neurones identified under green light and optogenetic stimulation of astrocytes using blue light, as previously described [9, 15]. Activation of RVLM astrocytes using flashing 470-nm light (<1 mW) depolarized C1 neurones (Fig. 2a). This depolarization of C1 neurones which followed optogenetic activation of astrocytes was strongly inhibited in the presence of an ATP degrading enzyme apyrase (25 U ml^−1^) (Fig. 2a; *P* < 0.01, Student’s *t* test), but was not affected by a P2Y_1_ receptor antagonist MRS2179 (10 μM; *P* = 0.68, Student’s *t* test) (Fig. 2b). These data indicate that release of ATP mediates excitation of presympathetic RVLM neurones in response to activation of local astrocytes. However, this effect does not appear to involve ATP binding to P2Y_1_ receptors which mediate astrocyte-triggered activation of neurones in the neighbouring retrotrapezoid nucleus as was shown previously using a similar experimental paradigm [15].

### Optogenetic stimulation of brainstem astrocytes in vivo increases sympathetic nerve activity, arterial blood pressure and heart rate

**Fig. 3:**
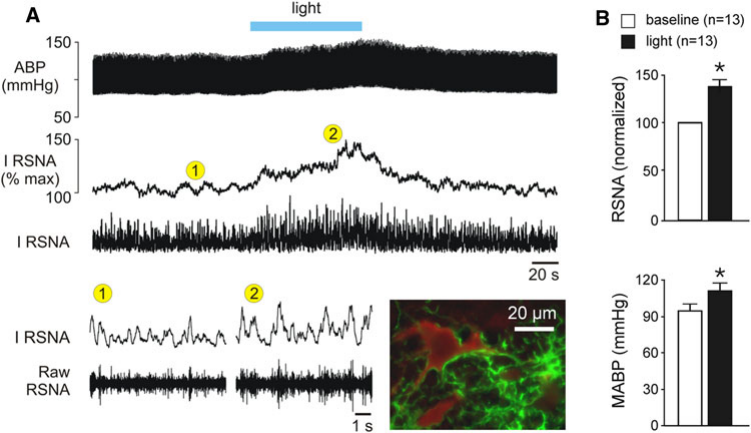
Optogenetic stimulation of RVLM astrocytes evokes sympathoexcitation in vivo. **a** Unilateral optogenetic stimulation of RVLM astrocytes expressing ChR2 increases sympathetic nerve activity and arterial blood pressure in anaesthetised and artificially ventilated rat. *ABP* arterial blood pressure, *IRSNA* integrated renal sympathetic nerve activity, *RSNA* renal sympathetic nerve activity. *Inset* microphotograph depicts an example of a tyrosine hydroxylase (red immunofluorescence)-expressing C1 neurone embraced by astrocytic processes expressing ChR2-Venus (green fluorescence). **b** Summary data illustrating the effect of optogenetic stimulation of RVLM astrocytes on mean arterial blood pressure (MABP) and RSNA. Group data are shown as mean ± SEM. *P < 0.05

Optogenetic stimulation of RVLM astrocytes expressing ChR2(H134R)-Katushka1.3 or ChR2-Venus (sympathetic and cardiovascular responses in both cases were similar and the data were pooled) resulted in a significant increases in renal SNA (by 36.5 %, *P* < 0.01; Student’s *t* test), mean arterial blood pressure (by 16.8 ± 2.7 mmHg, *P* < 0.01; Student’s *t* test), and heart rate (by 10.9 ± 2.35 bpm, *P* < 0.01; Student’s *t* test; *n* = 13) (Fig. 3a, b). These responses were sustained for the duration of the light stimulation (Fig. 3a). Histological analysis of the transduced brainstems revealed ChR2-expressing astrocytic processes surrounding and forming direct contacts with tyrosine hydroxylase-expressing presympathetic C1 neurones (Fig. 3a).

### Facilitated breakdown of ATP in the RVLM attenuates the progression of LV remodelling and heart failure secondary to myocardial infarction

Expression of TMPAP in the RVLM of naïve freely behaving rats had no effect on the arterial blood pressure and heart rate during the day or night phases of the 12-h/12-h circadian cycle (Supplementary Fig 1), although we could not test this in conscious rats with MI due to an unacceptable mortality rate.

**Fig. 4:**
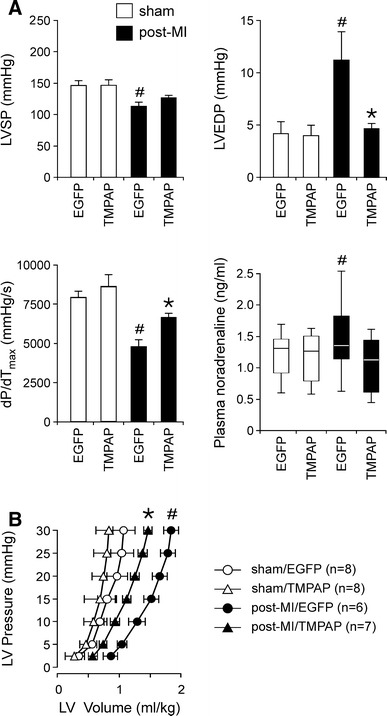
Expression of TMPAP in the RVLM attenuates the progression of left ventricular (LV) remodelling in a rat model of heart failure secondary to myocardial infarction (MI). **a** Summary data illustrating the effect of TMPAP expression in the RVLM on LV systolic pressure (LVSP), LV end diastolic pressure (LVEDP), maximum rate of LV pressure increase (d*P*/d*T*_max_) and noradrenaline concentration in blood plasma in rats with MI or sham-operated. Values are mean ± SEM except of noradrenaline data expressed as median and interquartile ranges. ^#^ *P* < 0.05 compared to values obtained in respective sham-operated animals. *P < 0.05 compared to values obtained in post-MI animals transduced to express EGFP in the RVLM. **b** In LV pressure–volume plots, *ash* denotes significant (*P* < 0.05) rightward shift of the LV pressure–volume relationship curve in post-MI animals transduced to express EGFP in the RVLM compared with the relationship curve in a respective sham-operated group. *Asterisk* indicates significant (*P* < 0.05) shift of the pressure–volume curve to smaller LV volumes in post-MI/TMPAP group compared with post-MI/EGFP group. *Numbers in parentheses* indicate sample sizes

The infarct sizes in groups of rats with MI transduced to express TMPAP–EGFP or EGFP were similar (27.3 ± 2.8 vs. 28.9 ± 3.4 %, respectively, *P* = 0.3; Table 1). Six weeks after MI, arterial blood pressure and heart rate (as measured under anaesthesia), heart weight, LV weight and pulmonary water content were similar in the groups of rats transduced to express TMPAP–EGFP or EGFP in the RVLM (Table 1). MI in rats transduced with LVV-EGFP into the RVLM led to the development of typical signs of LV remodelling and heart failure, characterised by reductions in LVSP (*P* < 0.05; ANOVA/Tukey–Kramer’s test) and dP/dT_max_ (*P* < 0.01; ANOVA/Tukey–Kramer’s test), elevation in LVEDP (*P* < 0.05; ANOVA/Tukey–Kramer’s test) (Fig. 4a) and a shift to the right of the LV pressure–volume relationship curve (*P* < 0.05; two-way ANOVA) (Fig. 4b). In rats with MI, bilateral expression of TMPAP in the RVLM maintained LVEDP (*P* < 0.01; ANOVA/Tukey–Kramer’s test), attenuated decline in dP/dT_max_ (*P* < 0.05; ANOVA/Tukey–Kramer’s test) and shifted the LV pressure–volume curve to the left (*P* < 0.05; two-way ANOVA) (Fig. 4a, b). Beneficial effect of TMPAP expression in the RVLM on LV function was accompanied by lower plasma concentration of noradrenaline (1.0 ± 0.2 ng/ml in the post-MI/TMPAP group vs. 1.6 ± 0.2 ng/ml in the post-MI/EGFP group; *P* < 0.05; Wilcoxon-Mann–Whitney *U* test) (Fig. 4a), indicative of a reduced sympathetic tone.

**Table 1:** Physiological parameters in animals with myocardial infarction-induced heart failure expressing either TMPAP or GFP in the rostral ventrolateral medulla

	Sham		Post-MI	
	EGFP (*n* = 8)	TMPAP (*n* = 8)	EGFP (*n* = 6)	TMPAP (*n* = 7)
Infarct size (%)			28.9 ± 3.4	27.3 ± 2.8
Body weight (g)	439 ± 16	422 ± 16	441 ± 14	432 ± 23
Heart weight (g)	1.4 ± 0.1	1.4 ± 0.1	1.9 ± 0.3*	1.9 ± 0.1*
LV weight (g)	0.9 ± 0.1	0.9 ± 0.1	1.2 ± 0.1*	1.2 ± 0.1*
Wet lungs weight (g)	1.7 ± 0.1	1.8 ± 0.1	2.1 ± 0.3	2.0 ± 0.3
Dry lungs weight (g)	0.4 ± 0.1	0.4 ± 0.1	0.5 ± 0.1	0.4 ± 0.1
Pulmonary water content (g)	1.2 ± 0.3	1.3 ± 0.1	1.6 ± 0.1	1.6 ± 0.2
Mean arterial blood pressure under anaesthesia (mmHg)	112 ± 3	113 ± 7	77 ± 5*	83 ± 3*
Heart rate (bpm)	355 ± 21	370 ± 13	343 ± 10	371 ± 15

*Bpm* beats per minute, *EGFP* enhanced green fluorescent protein (animals transduced to express EGFP in the rostral ventrolateral medulla oblongata), *LV* left ventricle, *MI* myocardial infarction, *TMPAP* transmembrane prostatic acid phosphatase (animals transduced to express TMPAP in the rostral ventraolateral medulla oblongata)

* *P* < 0.05 compared to values obtained in respective sham-operated animals

## Discussion

The present study suggests that in the developing heart failure, ATP-mediated purinergic signalling at the level of the RVLM contributes to sympathoexcitation and worsens LV function. This conclusion is supported by the data showing that facilitated breakdown of extracellular ATP in the RVLM in conditions of a virally driven expression of a potent ATP-degrading enzyme, TMPAP, leads to a reduction in plasma level of noradrenaline and attenuates the progression of LV remodelling and heart failure secondary to MI. Astrocytes are electrically non-excitable and communicate via release of ATP, and based on the existing evidence we postulate that astrocytes are the main source of extracellular ATP in the RVLM (as in the other parts of the brain). This hypothesis is supported by the evidence that cell-specific optogenetic stimulation of RVLM astrocytes activates presympathetic C1 neurones via an ATP-dependent mechanism.

This study was motivated by the clinical evidence of sleep-disordered breathing in heart failure with 27 % of patients experiencing episodes of obstructive sleep apnoea and 38 %, central sleep apnoea [3, 4]. Both syndromes are associated with recurrent periods of hypoxia which is capable of triggering release of ATP in the medulla oblongata [17]. Since heart failure patients with sleep apnoea have higher sympathetic tone [30, 32], we hypothesised that ATP actions at the level of the RVLM contribute to pathological sympathoexcitation and facilitate the progression of the disease. Indeed, application of ATP or ATP receptor agonists activates sympathoexcitatory RVLM neurones [37, 43] and leads to marked increases in the arterial blood pressure, heart rate and renal sympathetic nerve activity [23, 37, 43, 44].

Astrocytes release ATP in response to various stimuli and previously were shown to respond to hypoxia with elevations in intracellular Ca^2+^ [1]. It may be expected that such elevations lead to the release of ATP and even generation of ATP-mediated propagating Ca^2+^ waves using mechanisms similar to those described in our recent studies [15]. Here, we used an optogenetic approach for cell-specific stimulation of RVLM astrocytes and provide the first direct evidence that activated astrocytes are able to excite presympathetic C1 neurones via an ATP-dependent mechanism. This was demonstrated in organotypic brainstem slices containing the RVLM where sympathoexcitatory C1 neurones were genetically labelled with DsRed. Interestingly, while facilitated degradation of ATP in the presence of apyrase was highly efficient in blocking activation of C1 neurones which followed optogenetic stimulation of RVLM astrocytes, a P2Y_1_ receptor antagonist MRS2179 had no effect. In a similar experimental paradigm, MRS2179 was found to block activation of neurones in the nearby medullary retrotrapezoid nucleus [15] indicating that there are regional differences in the complement of purinergic receptors expressed by different groups of brainstem neurones.

Based on the existing evidence, it might be expected that optogenetic activation of RVLM astrocytes in vivo would trigger an ATP-mediated signalling pathway to C1 neurones. Indeed, we found that optogenetic stimulation of RVLM astrocytes leads to increases in renal sympathetic nerve activity, arterial blood pressure and heart rate. These results, when considered together with the literature data reporting increases in the arterial blood pressure and sympathetic nerve activity in response to microinjections of ATP into the RVLM, suggest that upon activation brainstem astrocytes may release ATP which serves as an excitatory signal to the presympathetic circuitry.

In order to evaluate the role of this mechanism in sympathoexcitation associated with progression of heart failure, we developed a molecular strategy for facilitated breakdown of ATP in the RVLM based on a virally driven expression and insertion into the cellular membranes of a potent ATP-degrading enzyme TMPAP. This molecular tool was first validated in cell cultures using a model of mechanical stimulation-induced propagation of astroglial Ca^2+^ excitation. Expression of TMPAP had a significant effect on Ca^2+^ responses in individual astrocytes and dramatically reduced the spread of Ca^2+^ excitation propagating among cultured astrocytes. Since Ca^2+^ ‘waves’ in astroglial cultures are known to be mediated via ATP release [2, 33], TMPAP expression appears to be highly effective in facilitating rapid breakdown of extracellular ATP.

We next evaluated the effect of TMPAP expression in the RVLM on cardiovascular homeostasis in healthy freely behaving animals. It was found that arterial blood pressure and heart rate were not affected by TMPAP expression in the RVLM during either day or night phase of the circadian cycle.

It is possible that in healthy animals other mechanisms can compensate for the reduction in purinergic tone in the RVLM. However, this signalling pathway may become particularly important in the developing heart failure since ATP release in the medulla oblongata increases in conditions of low *P*O_2_ [17]. A recent study using cerebral oxymetry has directly demonstrated that regional cerebral tissue oxygen saturation in many heart failure patients is significantly decreased despite nearly normal levels measured in the peripheral blood [38]. It is possible that this difference may become accentuated particularly during the episodes of sleep apnoea which are common in heart failure. There are several limitations to our study. First, we did not evaluate the effects of TMPAP expression in the RVLM on the absolute level of sympathetic nerve activity in freely behaving animals with MI. However, the validity of such measurements is questionable since differences in the contact between the nerve and the electrode inevitably result in differences in the signal amplitude and the signal-to-noise level. Second, due to technical limitations we were unable to measure cardiac- or total-body noradrenalin spillover. However, in our animals, TMPAP expression in the RVLM was accompanied by a significantly lower plasma concentration of noradrenalin which is typically elevated in rats with MI [25], and this is suggestive of a reduced sympathetic tone. We believe that measuring plasma levels of noradrenalin provides a good indicator of overall sympathetic nerve traffic, since most of plasma noradrenalin is derived from the sympathetic nerves (assuming the rate of plasma noradrenalin removal is similar in all our experimental groups [12]). It is also worth notice that in heart failure RVLM probably receives an enhanced input from the hypothalamus [24, 25] which may be further potentiated by local ATP-mediated signals. In this way, the summation of the two excitatory inputs within the RVLM could result in an excessive drive to the spinal sympathetic pre-ganglionic neurones.

Facilitated breakdown of ATP in the RVLM not only prevented an increase in plasma level of noradrenaline, but also attenuated the progression of LV remodelling in a rat model of heart failure secondary to MI. Indeed, expression of TMPAP bilaterally in the RVLM maintained normal LVEDP, attenuated decline in d*P*/d*T*_max_ and shifted the LV pressure–volume relationship curve to the left. Decreased d*P*/d*T*_max_ at normal LVEDP in post-MI rats transduced to express TMPAP in the RVLM could be explained by a concomitant slight increase in LV volume leading to normalisation of the LVEDP, as evident from the analysis of pressure–volume relationship (Fig. 4b). It is likely that further development of LV remodelling in this group would eventually lead to a progressive increase in LVEDP at a decreased LV contractility as described by [35]. Therefore, TMPAP expression in the RVLM might delay, but is unlikely to fully prevent the development of heart failure in the rat model used in this study. Since the expression of the transgenes driven by the lentiviral vectors similar to these used in this study requires at least 24 h, LVV-EF1α-TMPAP–EGFP and LVV-EF1α-EGFP were injected into the RVLM 2 days after inducing MI. This experimental design prevented TMPAP expression in the RVLM from having an effect on the acute ischemic injury (the infarct size was not affected) and sympathetic activity which might be crucial for survival in the period immediately following MI.

In summary, this study provides direct evidence that in developing heart failure actions of extracellular ATP within the RVLM presympathetic circuits contribute to increased sympathetic outflow, facilitate LV remodelling and worsen the development of heart failure. These results support commonly held view that sympathetic (over)activation in heart failure is maladaptive and detrimental, contributing to the progression of the disease. We hypothesise that astrocytes are the main source of extracellular ATP capable of increasing excitability of the RVLM presympathetic circuits. By extension, procedures or treatments which suppress astroglial activation and/or interfere with ATP-mediated signalling in brain regions regulating cardio-respiratory homeostasis might be expected to have a significant beneficial effect on the failing heart.

## Electronic supplementary material

Below is the link to the electronic supplementary material.

Supplementary material 1 (TIFF 216 kb)
