# Supplementary figures and images for: Purinergic signalling in the rostral ventro-lateral medulla controls sympathetic drive and contributes to the progression of heart failure following myocardial infarction in rats
Source: Basic Res Cardiol. 2012 Nov 28;108(1):317. doi: 10.1007/s00395-012-0317-x (PMC3540348; doi:10.1007/s00395-012-0317-x)

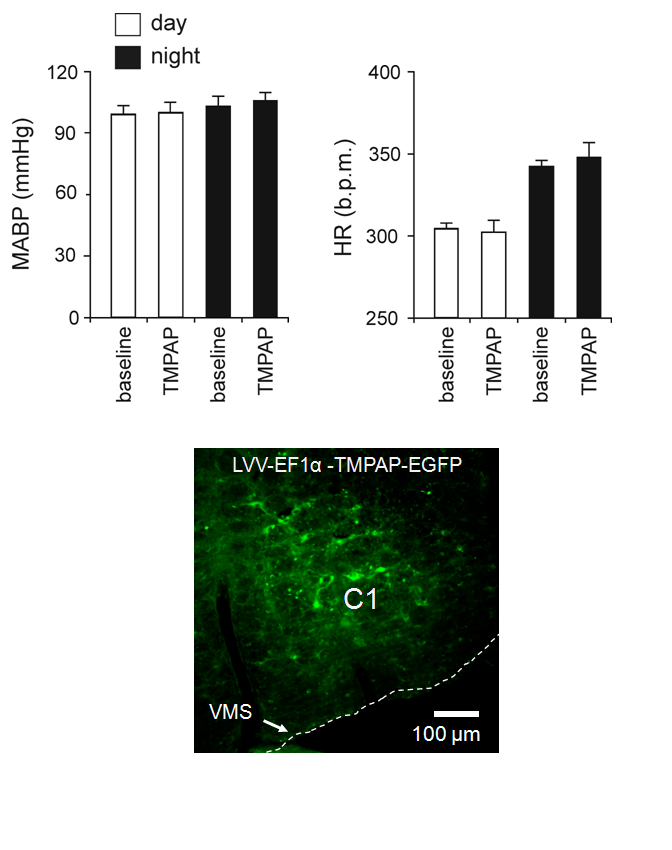

Supplement: Supplementary file 1 — Supplementary material 1 (TIFF 216 kb) [file 395_2012_317_MOESM1_ESM.tif]
